# Translation, cultural adaptation, and validation of the Japanese version of the quality of life assessment in spina bifida for adults

**DOI:** 10.1186/s41687-026-01018-z

**Published:** 2026-02-13

**Authors:** Tae Kawahara, Hitoshi Momose, Yoshifumi Sugita, Konrad M. Szymanski, Eisuke Hida, Akemi Yamazaki

**Affiliations:** 1 Department of Pediatric and Family Nursing, Division of Health Sciences, Graduate School of Medicine, The University of Osaka, Suita, Osaka Japan; 2 Department of Urology, Medical corporation Katsurakai HIRAO Hospital, Kashihara, Nara Japan; 3 Department of Urology, Kobe Children’s Hospital, Kobe, Hyogo Japan; 4 Division of Pediatric Urology, Riley Hospital for Children at Indiana University Health, Indianapolis, IN USA; 5 Department of Biostatistics & Data Science, Graduate School of Medicine, The University of Osaka, Suita, Osaka Japan

**Keywords:** Spinal dysraphism, Quality of life, Adult, Urinary incontinence, Fecal incontinence, Patient-reported outcome measures

## Abstract

**Background:**

The QUAlity of Life Assessment in Spina bifida (QUALAS) is a self-administered questionnaire that measures health-related quality of life. The Japanese versions of QUALAS for children and teenagers with spina bifida (SB) have been validated. This study aimed to develop and validate the reliability and validity of the Japanese version of QUALAS-A (QUALAS-A-J), the adult version of the instrument.

**Methodology:**

The participants were adults with SB aged ≥ 18 years. The results of cognitive interviews and a preliminary survey conducted on 16 participants were analyzed to confirm the face and content validity of the responses, and the item wording was modified. The revised questionnaire was administered from April to December 2022. The survey requested responses regarding demographics, QUALAS-A-J, and the World Health Organization Quality of Life Scale (WHOQOL-26). We then calculated descriptive statistics and correlation coefficients, and conducted exploratory factor analysis and Student’s *t*-test. Cronbach’s α and retests were used to determine reliability and intraclass correlation coefficients (ICCs), respectively.

**Results:**

Valid responses were received from 133 participants (52% female; mean age, 31.3 ± 10.5 years). Factor analysis indicated a 12-item, three-factor structure. Three items related to sexual activity that had low variance estimates were eliminated. Two factors converged on the same items as the original version; the correlation coefficients for QUALAS-A-J and WHOQOL-26 domains were 0.36 ≤ *r* ≤ 0.72, which confirmed discriminability for two domains. In all three domains, health-related quality of life was higher for those without than for those with urinary incontinence, validating known-groups validity. Cronbach’s α was 0.66–0.88 and the ICCs were > 0.8, thereby confirming reliability.

**Conclusions:**

The present study evaluated the reliability of QUALAS-A-J, which has three domains, 12 items, and two original and one new structure.

**Supplementary Information:**

The online version contains supplementary material available at 10.1186/s41687-026-01018-z.

## Abstract (plain language summary)

Adults with spina bifida (SB) in Japan have lacked a specific questionnaire to measure their health-related quality of life (HRQOL), which has hindered a complete understanding of their unique needs. Japanese versions of the QUAlity of Life Assessment in Spina bifida (QUALAS) questionnaire already exist for children and teenagers; developing a Japanese version of the QUALAS for adults (QUALAS-A) would complete this important age-specific series of assessments. Therefore, we developed and validated a Japanese version of the QUALAS-A questionnaire (QUALAS-A-J), a tool for assessing HRQOL in adults with SB, and then confirmed its reliability and cultural appropriateness (some sexuality-related questions were removed from the original QUALAS-A). The QUALAS-A-J is a trustworthy 12-item questionnaire with three key areas: “Health and Relationship”, “Esteem”, and “Bladder and Bowel”. This questionnaire completes a comprehensive set of HRQOL measures across all age groups and could serve as a valuable tool for health-care professionals in Japan by aiding the provision of improved assessments and support to adults with SB.

**Supplementary Information:**

The online version contains supplementary material available at 10.1186/s41687-026-01018-z.

## Background

Spina bifida (SB) is a neural tube closure defect that occurs early in pregnancy and presents various complications, including hydrocephalus, lower limb motor dysfunction, lower urinary tract dysfunction, anorectal disorders, and sensory palsy. SB has been found to have a 60% reduction in incidence with folic acid intake. Compared with Western countries, where folic acid intake has reduced SB incidence in recent years, the incidence of SB in Japan is 5∼6 per 1000 live births [1], showing no decreasing trend. This is partly due to Japan’s low intake of folic acid, at 18% [2], compared with Europe and the US, where 30%–50% of pregnant women consume folic acid before pregnancy [3–5].

After the acute neurosurgical management of SB immediately after birth, individuals with SB can start to lead a social life while managing various complications at home. With the early introduction of clean intermittent catheterization (CIC) for bladder management in the 1970s, the risk of renal failure was significantly reduced and long-term survival was expected [6, 7], and more than 90% of individuals with SB now reach adulthood. However, even though they reached adulthood in terms of age, individuals with SB accompanied by complex comorbidities still require a long time to transition completely to adult medical care [8].

It is a lengthy transition process for individuals with SB to acquire a variety of self-management skills because the successful maintenance of health and independence requires complex self-management, including planned and appropriate bladder and bowel management, regular hospital visits and medications, and balancing between social life and chronic disease status. Actually, in Japan, one of the patient-side causes of the stagnant transition to adult care among children with SB is that such individuals with concomitant bladder and bowel disorders do not progress independently, and complications arise resulting from a lack of regular medical visits [9]. Only about half of young adults with SB who require bladder and bowel management demonstrate adequate self-management [10], and as such, often fail to make a satisfactory transition to adult care. The concept of health-related quality of life (HRQOL), a patient self-report measure that includes chronic disease management, is an important assessment measure in regard to transition. However, general HRQOL scales that focus on physical health are insufficient to assess HRQOL accurately [11], and thus, self-report questionnaires that focus on health and psychosocial aspects specific to SB should be used.

In Japan, studies on the quality of life (QOL) of people with SB have been conducted using the five-level version of the EuroQol five-dimensional descriptive system [12] and a modified version of the Life Satisfaction Questionnaire for Elementary and Junior High School Students [13]. However, to our knowledge, no studies have used a cognitive HRQOL scale reflecting the characteristics of SB. Therefore, in the context of Japan, where disease-specific and psychosocial aspects of HRQOL in SB have not been sufficiently evaluated, there is a clear need for a validated, SB-specific instrument for adults.

Given this background, the present study focused on the QUAlity of Life Assessment in Spina bifida for Adults (QUALAS-A), a self-administered HRQOL scale [14] developed in the US for adults with SB aged ≥ 18 years. The QUALAS has also been developed for individuals aged 8–12 and 13–17 years (QUAlity of Life Assessment in Spina bifida for Children [QUALAS-C] and QUAlity of Life Assessment in Spina bifida for teenagers [QUALAS-T], respectively) [15, 16]. The QUALAS-A includes the same conceptual domain as the QUALAS-C/T, “Bladder and Bowel”, as well as the adult-specific domain of “Esteem and Sexuality”. Although the reliability and validity of the Japanese version of these scales, QUALAS-C/T-J, have already been confirmed [17, 18], it is important to develop a Japanese version of the QUALAS-A for assessment from a lifelong developmental perspective at longitudinal ages. The present study describes the development of an early-stage instrument to validate the QUALAS-A-J, intended for use in both descriptive research and longitudinal evaluations of treatment effects and transition support.

## Methods

### Aim

This study aimed to develop and evaluate the validity and reliability of a Japanese version of the QUALAS-A (QUALAS-A-J).

### Inclusion/exclusion criteria

We included adults with SB aged ≥ 18 years. The exclusion criteria were those who had not undergone spinal repair within the first year after birth, had serious conditions except for SB, had undergone surgery or hospitalization within the past month, had not received a confirmed diagnosis of SB, and were at a developmental stage not sufficient for a self-report response.

### Ethical considerations

The study protocol was approved by the Institutional Review Board of the University of Osaka (approval No. 21100, June 22, 2021). The work described herein was carried out in accordance with the Code of Ethics of the World Medical Association (Declaration of Helsinki) and the Ethical Guidelines for Medical and Biological Research Involving Human Subjects. All participants provided written informed consent through the pretest and main survey.

### Procedures

#### Phase 1: Preparation

In this cross-sectional study, we followed the Good Practice for the Translation and Cultural Adaptation Process of ISPOR [19], and developed the scale in accordance with the procedure requested by the original authors. First, we obtained permission from the original authors to develop the Japanese version of the scale. Next, we consulted with three urologists (one from the US), two nurses, a statistician, a pediatric neurosurgeon, an orthopedic surgeon, and two adults with SB from the SB outpatient clinic.

#### Phase 2: Forward-translation and coordination

Two urologists independently translated the QUALAS-A from English to Japanese. The team integrated the two translated versions. Items 11–15 were identical to the QUALAS-C/T, so these were adjusted to be expressed identically for instrument continuity (Version 1-a). The team reached a consensus on the wording of “sexual behavior” common to two items, and “having children” and “health problems” for one item, by adjusting the wording so that it was not unnatural to the Japanese language (Version 1-b).

#### Phase 3: Back-translation and review

Back-translation of Version 1-b was conducted by individuals fluent in both English and Japanese. The original authors on the team agreed to the Japanese translation of “sexual behavior and having children”, which was designated as Version 2.

#### Phase 4: preliminary survey and cognitive interview

A preliminary survey and cognitive interviews were conducted from September to December 2021. We recruited participants who met the inclusion criteria and could sufficiently respond to the questionnaire and cognitive interviews via video or telephone. Sampling was conducted by a neurosurgeon and nurse working, who collaborated for this study, working in an SB outpatient clinic, and 17 participants who were able to complete the QUALAS-A-J (Version 2) were selected to be interviewed. Survey forms were mailed to the participants’ homes, and survey responses and interviews were completed during a single videoconference. Response times and interviews were recorded in the preliminary survey; one participant was excluded from the analysis because of inconsistent responses. A skilled interviewer with experience in multiple scale development and qualitative research conducted interviews with all the participants to verify content and face validity from the perspectives of relevance, comprehensiveness, and clarity [20]. In a cognitive interview based on the verbal probing method, the participants were asked about the ease of responding, whether there were any difficult-to-understand sentences, if they felt uncomfortable answering, if the questionnaire adequately expressed their experiences, whether any questions seemed irrelevant, and if they had any other concerns. All interviews were recorded and transcribed verbatim. Sampling was terminated when the analysis of the transcripts showed saturation in the structure of the problems and potential solutions. Descriptive statistics were performed on the survey instrument, and trends in responses were examined in conjunction with the results of the interviews to revise the wording and content of each item for face and content validity (Version 2).

#### Phase 5: review of cognitive debriefing results

The content was also evaluated to confirm cultural equivalence. As a result, Version 3 was created. Compared with the US, sexual activity in Japan tends to be taboo, and many participants had difficulty understanding the meaning of “sexual activity”, so the Japanese version was annotated with sexual activity and examples were provided. Back-translation of Version 3 was conducted by individuals fluent in both English and Japanese (different from Phase 3). Version 3 was agreed upon by the team, including the original author. In Phase 6, Version 3 of the QUALAS-A-J was used.

#### Phase 6: main survey

The main survey was conducted from April to December 2022. In addition to the nationwide mailing method by the Japanese Spina Bifida Association, sampling was conducted by in-person distribution at two SB-related clinics in Osaka (urban) and Hokkaido (rural) to mitigate urban and rural bias. To calculate the intraclass correlation coefficient (ICC) as a reliability test, only the participants who received direct distribution were asked to participate in a retest 2 weeks later. The survey forms were distributed to 95 participants using the mailing method and 55 using the direct method; for the retest, survey forms were distributed to 39 participants. The completed questionnaires were either hand-delivered to the researcher or collected by mail. If the questionnaires were not returned within 1 month after mailing, the researcher sent a reminder, and those that were not collected were excluded. In addition, if more than 20% incomplete responses were received for each item, these were excluded from the analysis.

### Survey items

The participants were asked questions regarding their gender, age, education level, ambulatory status, shunt placing, CIC status, bladder enlargement, bowel management, employment status, and marital status.

#### The quality of life assessment in spina bifida for adults

The QUALAS-A [14] is a self-report HRQOL questionnaire for individuals with SB aged ≥ 18 years, following the QUALAS-C and QUALAS-T [15, 16]. The QUALAS-A is composed of 15 items in three domains: “Health and Relationships” (five items: able to do fun things, time with friends, close friendships outside family, overall health, and people saw you for more than health problems), “Esteem and Sexuality” (five items: bother by others helping, having future children, embarrassment about your look, bother by sexual in/activity, and future sexual satisfaction impacted by health), and “Bladder and Bowel” (five items: worry about pads being noticed, bother by bowel leak, bother by urine leak, urine problems stop you from fun things, and bother by waiting for bowel movement). All items are scored such that a higher score indicates a better HRQOL on a five-point Likert scale (from “not at all” = 0 to “always” = 20). Seven items included the response option “not applicable” (= 20) and sexuality-related items included the response option “do not want to answer” (= missing value). In the QUALAS series, “Not applicable” is scored as indicating a high HRQOL orientation, reflecting a lack of perceived problems [21]. This interpretation was also considered during the development of the Japanese version, and the same scoring approach was retained. This approach is applied consistently across the QUALAS instruments [14–16]. Each domain is scored independently from 0 to 100, with higher scores indicating higher HRQOL. Scoring for the QUALAS-A-J is in accordance with the QUALAS-A.

#### The world health organization quality of life scale

The World Health Organization Quality of Life Scale (WHOQOL-26) [22] is composed of 26 items in four domains: physical health (7 items), psychological health (6 items), social relationships (3 items), and environmental health (8 items). It also includes items on quality of life and general health. Each item on the WHOQOL-26 is scored on a scale from 1 to 5. The scores are then linearly transformed to a 0–100 scale. A Japanese version of the WHOQOL-26 was developed by Tazaki and Nakane [23] and has been validated for reliability and validity. Cronbach’s α in this study ranged from 0.63 to 0.88.

### Statistical analysis

Based on previous research [14], the sample size was calculated based on the following assumptions: (1) a factor analysis that requires five persons per item, that is, 75 persons; and (2) an alpha of 0.05, a power of 0.80, and a Pearson’s minimum value of discriminant validity of 0.39 for a two-tailed test using G * power [24], a cognitive interview sample size of at least seven persons for the assessment of content validity according to the COSMIN bias checklist, and at least 50 persons for a quantitative study (i.e., the main study) [25]. Consequently, it was determined that at least 46 participants were needed for this survey.

In the cognitive interviews, the transcribed verbatim records were analyzed using qualitative descriptive induction [26] to verify item phrasing problems and propose revisions in two stages. In the analysis, the first 12 participants were asked to speak freely about the scale items based on the interview guide, with the aim of reaching saturation on issues of content and face validity. The next four participants were asked not only to speak freely, but also to answer questions about a systematic set of identified problems and revisions, thereby providing feedback on the proposed revisions. The revision proposals were then shared with the team in both Japanese and English.

All responses were statistically processed using SPSS for Windows (version 28; IBM, Tokyo, Japan). The continuous data items were evaluated for a normal distribution using the Shapiro–Wilk test, a Q–Q plot, and histograms. Descriptive statistics were performed for all attributes and items/domains. Discriminant validity was determined by comparing the strength of the correlation coefficients between the QUALAS-A-J and WHOQOL-26 domains. Correlation coefficients were calculated using the following cutoff values: *r* < 0.20 = very weak; 0.20–0.39 = weak; 0.40–0.59 = moderate; 0.60–0.79 = strong; and 0.80–1.0 = very strong [27].

An exploratory factor analysis was conducted to assess construct validity. As in the original version, we conducted a principal factor analysis with varimax rotation, and set the factor eigenvalue > 1.0. The sample validity of the factor analysis was calculated using a Kaiser–Meyer–Olkin (KMO) coefficient > 0.60 [28].

For known-groups validity, we hypothesized the following for the domains obtained through exploratory factor analysis and conducted *t*-tests: In the Health and Relationship, Esteem, and Bladder and Bowel domains, those without urinary incontinence would have a higher score than those with urinary incontinence [29–31].

In this study, we tested for differential item functioning (DIF; McFadden’s R^2^ < 0.02) [25] across gender and age within the obtained factor structure using an ordinal logistic regression analysis. As a reliability test, Cronbach’s α, an internal consistency index, was calculated for each domain of the QUALAS-A-J. In addition, the number of ICCs between this study and the retest QUALAS-A-J was calculated as the test–retest reliability. Reliability was considered confirmed at > 0.7 for both [32, 33]. P-values ≤ 0.05 were considered statistically significant.

## Results

### Preliminary survey

In total, there were five male and 11 female respondents (mean age, 25.8 ± 4.6 years). The average response time was 3 min and 40 s (range, 1–10 min). For items 8–10 related to sexuality, three respondents (19%) each chose “do not want to answer”. Nine items (Nos. 1, 3–6, 11, 12, 14, and 15) were considered to be satisfactory in expression and content by all interviewees. Items that had problems with wording included, “I was not sure whether to answer that ‘health problems’ were due to spina bifida or to the overall physical condition”, “The phrases ‘sexual activity’ and ‘having children’ are difficult to understand”, and “It is difficult to understand what is meant by ‘having fun’”. Based on these comments, the team discussed the issues and modified the wording of the document to explain that “health problems” referred to overall health, including SB. In addition, the items related to sexuality were revised with the addition of examples and expressions, and “having fun” was changed to age-appropriate Japanese wording.

### Main survey

Of the 150 participants, 133 (valid response rate: 89%) were included in the analysis. The excluded participants included 10 who did not return the survey, one who refused to respond, two whose developmental stage made it difficult for them to respond, and four who had undergone spinal cord surgery more than 1 year after birth. Of the 39 surveys distributed for the retest, 24 were included in the analysis. We excluded four participants who did not return the survey and 11 who responded outside the time frame, leaving a total of 15 responses for analysis.

#### Demographic characteristics

Table 1 shows the demographic characteristics of the respondents. The study participants were 51.9% female, with a mean age of 31.3 ± 10.5 years (age range, 18–69 years). In addition, 45.1% had a high school-level education, 40.6% reported having an employment status of full-time, and 18.1% were married or had a partner. Furthermore, 60.9% reported having ambulatory walking status, 36.8% a ventriculoperitoneal shunt, 81.2% CIC, and 72.9% bowel management.

**Table 1:** Characteristics of the participants in the present study

	*n* or mean	% or SD
Gender		
Male	64	48.1
Female	69	51.9
Age (years)	31.3	10.5
Education background		
Junior high/high school	60	45.0
Junior college/vocational school	18	13.6
University	37	27.8
Graduate school	5	3.8
Other	5	3.8
Mobility		
Community ambulator	81	60.9
Household ambulator	7	5.3
Non-ambulator	41	30.8
Ventriculoperitoneal shunt		
Yes	49	36.8
No	78	58.6
Uncertain	4	3.0
Employment status		
Full-time	54	40.6
Contract employee	15	11.3
Part-time	12	9.0
Self-employed	2	1.5
Student	9	6.8
Unemployed	24	18.0
Marital status		
Never married (no partner)	103	77.4
Never married (with a partner)	5	3.8
Married	19	14.3
Divorced or bereaved	3	2.3
Clean intermittent catheterization		
Yes	108	81.2
No	24	18.0
Primary method of bowel management		
Enema	8	6.0
Laxatives	20	15.0
Defecation	26	19.5
Anal plugs	2	1.5
Transanal irrigation	29	21.8
Antegrade continence enema	10	7.5
Other	3	2.3
WHOQOL-26		
Physical health	23.5	5.7
Psychological health	19.4	5.5
Social relations	9.6	2.4
Environment	28.6	5.8

SD = standard deviation; WHOQOL-26 = World Health Organization Quality of Life Scale

#### Ceiling and floor effects and missing data

The ceiling effect was confirmed by items 7 (“Bother by others helping”) and 9 (“Bother by sexual in/activity”), with mean scores + standard deviation of 15.8 + 6.0 and 14.2 + 6.7, respectively (data not shown). Regarding item 7, 41.4% of the respondents selected “not applicable”, which was scored as the maximum value and therefore contributed to the ceiling effect. By contrast, regarding item 9, 35.3% of the respondents chose “do not want to answer”; these responses were treated as missing data. In addition, 15.8% and 24.1% of the respondents did not want to answer items 8 (“Embarrassment about your look”) and 10 (“Future sexual satisfaction impacted by health”), respectively. No items indicated a floor effect.

#### Factor analysis and cross-cultural validity/measurement invariance

Factor analysis with all 15 items yielded a KMO coefficient of 0.75. Four factors with initial eigenvalues > 1 were extracted, with items 1–5 classified as factor 1, items 6 and 7 as factor 2, items 8 and 10–13 as factor 3, and items 14 and 15 as factor 4; however, item 9 had a factor loading of < 0.4 and was not classified as any factor, and the meaning of the classified factor could not be explained in comparison with the original version. A 14-item factor analysis eliminating item 9 yielded a KMO coefficient of 0.77 and four factors with initial eigenvalues > 1, but the eigenvalue of factor 4 was as small as 1.06, so the number of factors was fixed at three and varimax rotation was performed. The results showed that items 1–5 were classified as factor 1, items 6 and 7 as factor 2, and items 8 and 10–15 as factor 3. Items 8 and 10, which converged to factor 2 in the original version, converged to factor 3 in the present study. In the factor analysis of the 13 items excluding items 9 and 10, item 8 was also classified as factor 3, indicating a factor structure different from that of the original version.

Therefore, due to the characteristic of the factor analysis of being affected by the estimated variance, a factor analysis was conducted with 12 items, excluding items 8–10, whose precision of variance estimation was lower than that of the other items, and the KMO coefficient was 0.80. Three factors with initial eigenvalues > 1 were extracted, with items 1–5 converging to factor 1, items 6 and 7 to factor 2, and items 11–15 to factor 3. Although the factor loadings for item 2 indicated that factors 1 and 2 were similar in magnitude, the QUALAS-A-J was classified as factor 1 in accordance with the original version because the QUALAS is an age-related instrument and the QUALAS-C/T-J converged on factor 1 (Table 2). This factor structure was the same as that of the original version. Factors 1 and 3 were designated “Health and Relationship” and “Bladder and Bowel”, respectively, as in the original version, and factor 2 was named “Esteem”. A total of 12 items and a three-domain structure were determined as the final version of the QUALAS-A-J. This domain structure was approved by the entire team, including the original author.

**Table 2:** Exploratory factor analysis with 12 items on the QUALAS-A-J

		Factor 1	Factor 2	Factor 3	Extraction
1	Able to do fun things	**0.43**	0.38	0.38	0.47
2	Time with friends	**0.42**	0.43		0.39
3	Close friendships outside family	**0.69**			0.61
4	Overall health	**0.72**			0.52
5	People saw you for more than health problems	**0.86**			0.77
6	Embarrassment about your look		**0.60**	0.53	0.67
7	Bother by others helping		**0.59**		0.37
11	Worry about pads being noticed			**0.66**	0.49
12	Bother by bowel leak			**0.80**	0.68
13	Bother by urine leak			**0.80**	0.65
14	Urine problems stop you from fun things			**0.57**	0.37
15	Bother by waiting for bowel movement			**0.50**	0.31

Factor analysis was explained by the maximum likelihood method with varimax rotation. Whole items of QUALAS-A-J are quotes from Szymanski et al. [12]. Items with a factor loading > 0.35 are shown. The Kaiser–Meyer–Olkin (KMO) coefficient was 0.80 and the cumulative contribution ratio of the three factors was 63.9%. QUALAS-A-J = Japanese version of the QUAlity of Life Assessment in Spina bifida for Adults.

#### Scoring of the QUALAS-A-J

In the QUALAS-A-J, the scores for “Health and Relationship” and “Bladder and Bowel” both range from 0 to 100 points. Because “Esteem” was reduced to a two-item domain, we applied a standard practice of linear rescaling to a common metric to ensure comparability across domains. The score is multiplied by 2.5 to give a score from 0 to 100 for comparison with the other domains. Descriptive statistics for the QUALAS-A-J are shown in Table 3. There were no missing values in any domain, with ICCs > 0.82 and Cronbach’s α ranging from 0.66 to 0.81. The result of the DIF analysis on the new domain structure showed that McFadden’s R^2^ was < 0.013 for age and gender.

**Table 3:** Scores for scales on the QUALAS-A-J

Domain	*n*	Mean (SD)	Median (range)	% scoring minimum	% scoring maximum	Cronbach’s α	Test–retest reliability (ICC)
Health and Relationship	133	58.0 (22.9)	60.0 (0.0–100.0)	0.8	2.3	0.81	0.82
Esteem	133	57.7 (27.0)	25.0 (0.0–100.0)	3.8	10.5	0.66	0.83
Bladder and Bowel	133	55.6 (24.9)	55.0 (0.0–100.0)	3.8	3.8	0.81	0.89

QUALAS-A-J = Japanese version of the QUAlity of Life Assessment in Spina bifida for Adults; SD = standard deviation; ICC = intraclass correlation coefficient

#### Discriminant validity

The domain correlation coefficients between the QUALAS-A-J and WHOQOL-26 are shown in Table 4. Regarding the correlation coefficients between the QUALAS-A-J domains, the correlation between the “Health and Relationship” and “Bladder and Bowel” domains, which have the same items as the original version, was small, at *r* = 0.35, while that between the “Health and Relationship” and “Esteem” domains was a large positive correlation, at *r* = 0.78. The correlation coefficients between the WHOQOL-26 and the “Esteem” and “Bladder and Bowel” domains were weak (0.59 ≤ *r* ≤ 0.67, and 0.36 ≤ *r* ≤ 0.53, respectively), which indicate that these two domains reflect different aspects of the WHOQOL-26. The correlation coefficient for the “Health and Relationship” domain was strong, but that between “Health and Relationship” and the WHOQOL-26 in the original version was 0.63 ≤ *r* ≤ 0.71, which is similar to that in the present study. Therefore, to confirm construct validity within the three-domain structure, we verified known-groups validity.

**Table 4:** Correlation matrix between the QUALAS-A-J and WHOQOL-26

	Health and Relationship	Esteem	Bladder and Bowel
Esteem	.78^a^		
Bladder and bowel	.35^a^	.52^a^	
**Total WHOQOL-26**	.67^a^	.59^a^	.53^a^
Physical health	.68^a^	.64^a^	.43^a^
Psychological health	.72^a^	.67^a^	.46^a^
Social relationships	.64^a^	.62^a^	.53^a^
Environmental health	.68^a^	.61^a^	.36^a^
			(*N* = 131∼133)

QUALAS-A-J = Japanese version of the QUAlity of Life Assessment in Spina bifida for Adults; WHOQOL-26 = World Health Organization Quality of Life Scale. ^a^ *p* < 0.01

#### Known-groups validity

To examine the known-groups validity of the three domains of the QUALAS-A-J, we conducted Student’s *t*-test for each domain (Health and Relationship, Esteem, and Bladder and Bowel) based on the presence or absence of urinary incontinence. As shown in Table 5, the hypothesis was confirmed in each domain.

**Table 5:** Independent samples *t*-test for known-groups validity

Variables	No urinary incontinence			Urinary incontinence				
	*n*	Mean	SD	*n*	Mean	SD	t-value	*p*-value
Health and Relationship	32	66.1	22.4	101	55.4	22.5	2.3	0.02
Esteem	32	28.0	7.8	101	21.5	11.2	3.6	0.001
Bladder and Bowel	32	69.7	19.9	101	51.1	24.7	4.3	0.0001

QUALAS-A-J = Japanese version of the QUAlity of Life Assessment in Spina bifida for Adults; SD = Standard deviation

## Discussion

In the present developmental validation study, we formed a team of experts from different professions to examine the content and face validity, construct validity, and reliability of the QUALAS-A-J for adults with SB in Japan using forward-translation, back-translation, a preliminary survey, and cognitive interviews. The findings indicated that the QUALAS-A-J has acceptable cultural and content validity and reliability, a short response time of 5 min [34], and can be classified into 12 items and three domains.

In the present study, the sample size (*N* = 133) was large for a Japanese SB study, approximately 75% of the participants were employed, and the age and gender distribution can be said to constitute a representative population of adults with SB who have a social life. The age of the participants in this study was similar to the distribution in the original version (32.1 ± 11 years) [14], and the male to female ratio was 1:1, which resulted in a well-balanced sample. Furthermore, the face validity of the instrument was validated by the results of the cognitive interviews, the short response time in the preliminary survey, and the high response rate for the QUALAS-A-J in the main survey. We adapted the original QUALAS-A to the needs of the Japanese population based on qualitative and quantitative patient-reported data. A similar approach was used in German and Korean validation studies of the QUALAS [35, 36].

Factor analysis of QUALAS-A-J revealed an underlying 3-factor structure with factor loadings similar to those in the original QUALAS-A. Despite an abbreviated “Esteem” domain and administration in a different population, the underlying structure of the QUALAS-A-J is similar to the original. The results of the factor analysis revealed that the “Health and Relationship” and “Bladder and Bowel” domains had the same structure as the original version, indicating that these domain structures could be used across age groups. On the other hand, about 30% of the participants chose “do not want to answer” for the items on sexual activity, so three items related to sexual activity were omitted from the factor analysis. The finding that all items related to sexuality were deemed inapplicable is considered to be the result of two factors: the repressive cultural background toward sexual behavior in Japan, and the insufficient provision of sexual education for individuals with SB. In a study on sexual behavior conducted in Japan in a population aged 20–50 years [37], the percentage of both men and women who reported having no partner in regard to sexual activity was 45% for each, a higher percentage than that in the US (28%) [38], and 37.4% of women and 54.2% of men in Japan reported that sexual activity was important [37], which are lower than the percentages reported in countries such as the UK [39] and France [40]. In addition, the percentage of respondents in Japan who reported never having had sexual intercourse in their lifetime increased from 2015 to 2022, indicating that Japanese people tend to be less sexually active than their counterparts in Western countries [37]. In addition to the repressive cultural background toward sexual behavior in Japan, the sexuality of people with disabilities in Japan is considered taboo [41], and it is clear that sexual education for people with SB is not sufficient to provide evidence [42]; therefore, individualized sexual education programs, especially for Japanese people with SB, remain a challenge [43]. This may have limited the responses to the items related to sexual activity compared with the original version, resulting in lower values for extraction in the factor analysis. In other words, some of the items pertaining to sexuality in the original version were not included in the QUALAS-A-J to improve its clinical usefulness and acceptability for Japanese adults with SB. However, numerous studies have reported that sexual activity influences HRQOL. In Japan, topics related to sexual activity are becoming increasingly important to individuals with SB, so sexual activity specific to Japan needs to be clarified through cognitive interviews and examined in more detail, and the addition of items to the QUALAS-A-J needs to be considered. In the present study, DIF was examined within the domain structure, and because the MacFoster R^2^ values for gender and age were both < 0.02, no DIF was observed, thereby confirming cross-cultural validity and measurement invariance.

Only one item (item 7) appeared to have a ceiling effect, as a high percentage of respondents indicated they were not given special treatment. This may be due to the fact that SB is a congenital disease and patients rely on caregivers for routine medical care that continues after birth, thereby normalizing the interdependent relationship between parents and children [44]. Furthermore, parents rearing children with chronic illnesses tend to be over-involved with their own children during puberty, as children begin the transition to self-management, and thus, their children may perceive a helping environment as normal [45, 46] and carry this perception into adolescence. In Japan, in particular, there is a strong tendency to depend on parents even beyond adolescence [47], which may explain why a higher percentage of Japanese adults with SB in this study took parental support for granted from childhood and perceived that they were not treated specially.

The strong correlation observed between the QUALAS-A-J “Health and Relationship” and “Bladder and Bowel” domains was similar to that in the original version. On the other hand, the correlation between “Health and Relationship” and “Esteem” was also strong. Previous research on esteem has suggested that self-esteem and social relationships are truly interactive across the life-span, reflecting a positive feedback loop between both constructs [48]. Moreover, a study of young adults with chronic illnesses found that self-esteem and changes in the perception of health status are closely related [49]. From these findings, it can be inferred that the correlation between the “Esteem” and “Health and Relationship” domains without the sexuality factor in this study resulted in strong correlation coefficients because of the complementary relationship of the concepts to each other. In the assessment of known-groups validity, the hypotheses were confirmed for each domain structure, demonstrating that the scale can be specifically applied to the Japanese population with SB.

### Limitations

This study has several limitations. First, the overall sample size limited some aspects of the psychometric evaluation. In particular, the retest sample was relatively small (*n* = 15), which may restrict the precision of the stability estimates. Moreover, although exploratory factor analysis provided preliminary evidence of construct validity, the available sample was not sufficient to conduct a robust confirmatory factor analysis, especially given the multi-domain structure of the instrument. These limitations partly reflect the impact of the COVID-19 pandemic, during which, in-person survey distribution was constrained, resulting in fewer participants than initially planned and making systematic sampling difficult. Consequently, the evidence for construct validity remains preliminary, and future studies with larger, independent samples are warranted to replicate the factor structure using confirmatory factor analysis, and to further confirm the temporal stability of the scale. In addition, because this study employed a cross-sectional design, it was not possible to evaluate fully certain measurement properties recommended in the COSMIN Risk of Bias checklist [24], such as responsiveness to change and measurement errors. Future research should therefore include longitudinal designs with larger sample sizes to assess responsiveness and sensitivity to change, as well as to establish construct validity further.

Second, we distributed the survey in person at SB-related clinics to target a population that may be more interested in HRQOL. Such characteristics of the study population may have elevated overall HRQOL levels and could have introduced potential bias in the examination of discriminant validity with the WHOQOL-26. However, it is important to note that there may be a certain number of adults with SB who have low compliance and do not belong to an association or attend regular clinics for individuals with SB. To recruit such a target group, it would be necessary to invite them to participate through social networking services and consider allowing them to respond casually to not only paper-based, but also a web-based survey in future studies.

Third, we did not perform a gender-based analysis, as our primary objective was to validate the overall reliability and validity of the QUALAS-A-J. Our sample size, while sufficient for the main analysis, was not large enough for robust gender subgroup comparisons. Future research should explore potential gender differences in HRQOL among Japanese adults with SB.

## Conclusion

In the present study, we evaluated the reliability and validity of the QUALAS-A-J as a 12-item, three-factor instrument with expressions that are easily understood by Japanese adults with SB.

## Supplementary Information

Below is the link to the electronic supplementary material.

Supplementary Material 1

## Data Availability

The datasets used and/or analyzed during the current study are available from the corresponding author on reasonable request.

## Abbreviations

SB: Spina bifida
CIC: Clean intermittent catheterization
HRQOL: Health-related quality of life
QUALAS-A: QUAlity of Life Assessment in Spina bifida for Adults
QUALAS-C-J: Japanese version of the QUAlity of Life Assessment in Spina bifida for Children
QUALAS-T-J: Japanese version of the QUAlity of Life Assessment in Spina bifida for Youth
ICC: Intraclass correlation coefficient
WHOQOL-26: World Health Organization Quality of Life Scale
KMO: Kaiser–Meyer–Olkin
SD: Standard deviation
DIF: Differential item functioning
