# Supplementary material for: Translation, cultural adaptation, and validation of the Japanese version of the quality of life assessment in spina bifida for adults
Source: J Patient Rep Outcomes. 2026 Feb 13;10:41. doi: 10.1186/s41687-026-01018-z (PMC13004760; doi:10.1186/s41687-026-01018-z)
Supplement: Supplementary file 1 — Supplementary Material 1 [file 41687_2026_1018_MOESM1_ESM.docx]

**Plain English Summary**

Adults with spina bifida (SB) in Japan have lacked a specific questionnaire to measure their health-related quality of life (HRQOL), which has hindered a complete understanding of their unique needs. Japanese versions of the QUAlity of Life Assessment in Spina bifida (QUALAS) questionnaire already exist for children and teenagers; developing a Japanese version of the QUALAS for adults (QUALAS-A) would complete this important age-specific series of assessments. Therefore, we developed and validated a Japanese version of the QUALAS-A questionnaire (QUALAS-A-J), a tool for assessing HRQOL in adults with SB, and then confirmed its reliability and cultural appropriateness (some sexuality-related questions were removed from the original QUALAS-A). The QUALAS-A-J is a trustworthy 12-item questionnaire with three key areas: “Health and Relationship”, “Esteem”, and “Bladder and Bowel”. This questionnaire completes a comprehensive set of HRQOL measures across all age groups and could serve as a valuable tool for health-care professionals in Japan by aiding the provision of improved assessments and support to adults with SB.
